# An Intervention to Increase Condom Use Among Users of Chlamydia Self-Sampling Websites (Wrapped): Intervention Mapping and Think-Aloud Study

**DOI:** 10.2196/11242

**Published:** 2019-05-01

**Authors:** Katie Newby, Rik Crutzen, Katherine Brown, Julia Bailey, John Saunders, Ala Szczepura, Jonny Hunt, Tim Alston, S Tariq Sadiq, Satyajit Das

**Affiliations:** 1 Centre for Advances in Behavioural Science Faculty of Health and Life Sciences Coventry University Coventry United Kingdom; 2 Health Promotion School for Public Health and Primary Care Maastricht University Maastricht Netherlands; 3 Primary Care and Population Health Institute of Epidemiology and Health University College London London United Kingdom; 4 Health Protection Services Public Health England London United Kingdom; 5 Enterprise and Innovation Coventry University Coventry United Kingdom; 6 Going Off The Rails Coventry United Kingdom; 7 Preventx Sheffield United Kingdom; 8 Institute for Infection and Immunity St George's, University of London London United Kingdom; 9 Coventry and Warwickshire Partnership Trust Coventry United Kingdom

**Keywords:** sexually transmitted infection, condoms, sexual behavior, young adult, intervention development, internet, eHealth, co-design

## Abstract

**Background:**

Young people aged 16-24 years are disproportionately affected by sexually transmitted infections (STIs). STIs can have serious health consequences for affected individuals and the estimated annual cost of treatment to the National Health Service is £620 million. Accordingly, the UK government has made reducing the rates of STIs among this group a priority. A missed opportunity to intervene to increase condom use is when young people obtain self-sampling kits for STIs via the internet.

**Objective:**

Our aim was to develop a theory-based tailored intervention to increase condom use for 16-24-years-olds accessing chlamydia self-sampling websites.

**Methods:**

The intervention, Wrapped, was developed using Intervention Mapping and was co-designed with young people. The following steps were performed: (1) identification of important determinants of condom use and evidence of their changeability using computer and digital interventions; (2) setting the intervention goal, performance objectives, and change objectives; (3) identification of Behavior Change Principles (BCPs) and practical strategies to target these determinants; and (4) development of intervention materials able to deliver the BCPs and practical strategies.

**Results:**

Users of existing chlamydia self-sampling websites are signposted to Wrapped after placing an order for a sampling kit. Salient barriers to condom use are identified by each user and relevant intervention components are allocated to target these. The components include the following: (1) a sample box of condoms, (2) an online condom distribution service, (3) a product for carrying condoms, (4) a condom demonstration video, (5) a series of videos on communication about condom use, and (6) erotic films of real couples discussing and demonstrating condom use.

**Conclusions:**

This intervention will be directed at young people who may be particularly receptive to messages and support for behavior change due to their testing status.

## Introduction

The UK Department of Health has made reducing the rates of sexually transmitted infections (STIs) a priority, particularly among young people who are disproportionately affected [1]. STIs can lead to serious health consequences, such as pelvic inflammatory disease, ectopic pregnancy, and infertility, which have a significant impact on quality of life [2]. The estimated annual cost to the National Health Service of STI treatment is £620 million [3]. One of the most effective ways to avoid STIs is to use a condom during penetrative sex [1] but young people report inconsistent use [4,5]. A missed opportunity to intervene to increase condom use is when young people obtain self-sampling kits for STIs via the internet.

As part of the National Chlamydia Screening Programme (NCSP) [6], there are a number of websites offering free self-sampling kits to young people living in England for the STI *Chlamydia trachomatis*. These kits are used by young people to collect a specimen, which is sent to a laboratory for testing. Laboratories then send the test results back to individuals. A total of 132,000 15-24-year-olds were tested via this route in 2017, representing once again an increase in use from the previous year [7]. Those tested are at high risk of acquiring STIs and include groups that other services, such as general practice and community sexual and reproductive health services, have found difficult to engage [8]. These websites therefore provide a unique opportunity to intervene with a priority population; however, they typically provide little or no sexual health promotion [8] despite the personal relevance of testing, making this an ideal “teachable moment” [9].

Digital health behavior-change interventions targeting young people have good potential reach. Access to the internet by young people living in the United Kingdom is now almost universal, with 99% of 16-24-year-olds reporting that they use the internet at home or elsewhere [10]. Digital interventions also offer a number of advantages to users over face-to-face delivery, including enabling access to content anonymously, repeatedly, and at convenient times [11,12]. The relatively low running costs of digital interventions following development [13] and their potential to deliver tailored content to individual users [14] with high levels of fidelity [15] also make them attractive to developers. Systematic reviews seeking to establish the efficacy of digital interventions for increasing protective sexual behavior have so far, however, only identified small effects (Cohen *d*<0.3) [16,17]. In order to harness their potential, it is necessary to identify efficacious content and characteristics.

Digital interventions are more effective at changing sexual behavior when tailored than nontailored [17]. Tailoring content often requires users to respond to multiple survey-style questions prior to receiving the intervention. It is essential, however, that tailoring methods used in digital interventions are acceptable, particularly in terms of burden, otherwise users in the real world will disengage. One parsimonious approach asks users to simply self-select from a predefined list the most important determinants of their behavior and to present content to match these. This has been identified as a reliable technique for isolating important behavioral determinants [18-20] but, to date, this approach has not been applied within a digital sexual health intervention [21].

This article describes the development of a tailored behavior change intervention, *Wrapped*, for increasing condom use among 16-24-year-olds using chlamydia self-sampling websites. The aim of the intervention is to reduce the incidence of STIs in this at-risk population. To the authors’ knowledge, this is the first digital sexual health intervention developed specifically for young people accessing STI self-sampling websites.

## Methods

### Overview

Wrapped was developed using Intervention Mapping [22]. Intervention Mapping provides a framework for the development of theory- and evidence-based interventions and is consistent with Medical Research Council guidance on the development of complex interventions [23]. It involves a number of sequential and iterative steps. Steps 1-4 relate to intervention development per se; the purpose and outcomes of these steps are described in Table 1 below. Steps 5 and 6 relate to planning for implementation, adoption, and evaluation. These plans are described at the end of the Results section.

Throughout steps 1-4, a combination of primary research, secondary research, and co-design methods were used to determine and refine intervention content.

### Step 1: Needs Assessment

The research team was made up of three health psychologists (KN, KB, and RC) and a sexual health doctor (JB), all with considerable sexual health research experience. The team consulted a number of sources of evidence in order to select determinants to be targeted by the intervention. Firstly, a review of reviews [24] reporting on the strength of association between condom use and behavioral determinants was examined. Determinants of condom use with a correlation above .2, corresponding to a small-to-medium effect size, were considered to be important and were selected. Secondly, three recent meta-analyses examining the effect of digital interventions on the determinants of health behavior [21,25,26] were consulted to establish the changeability of the selected determinants.

**Table 1:** Intervention Mapping steps, their purpose, and their outcomes.

Intervention Mapping step	Purpose	Outcome
1. Needs assessment	To identify what the intervention should address	Documented behavioral determinants
2. Intervention outcomes and objectives	To clarify the intervention goal and performance objectives, and to identify the immediate change objectives that need to be achieved in order to realize the intervention goal	Intervention goal and performance objectives, and matrices of change objectives
3. Intervention design	To identify Behavior Change Principles linked to change objectives and translate them into practical applications that are most likely to bring about the desired behavioral change via the identified determinants	Documented Behavior Change Principles linked to change objectives and their translation into practical applications
4. Intervention production	To develop and finalize the intervention structure and content	The final intervention

### Step 2: Intervention Outcomes and Objectives

#### Establishing Intervention Goal and Performance Objectives

The intervention goal was discussed and agreed upon by the study steering group. The steering group consisted of the above research team as well as three additional sexual health doctors (STS, JS, and SD), a professor of health technology assessment (AS), a sex education consultant (JH), and the technical director of an STI diagnostics business, which runs an STI self-sampling website (TA). Subsequently, the research team broke the intervention goal down into performance objectives, specifying what was required of intervention participants to achieve this goal. These were approved and finalized by the steering group.

#### Developing the Matrix of Change Objectives

A matrix was developed by the research team by combining the performance objectives and associated determinants to create change objectives (ie, positive statements about what needs to happen in order for the performance objectives to be achieved). To inform the selection of change objectives, the review of reviews that was previously consulted to identify important determinants to target with the intervention [24] was further examined. Beliefs identified as having a correlation above .2 were selected and used to inform specification of change objectives where relevant. Change objectives were discussed, refined, and agreed upon by the research team.

### Step 3: Intervention Design

#### Selection of Behavior Change Principles

The term *Behavior Change Principle* (BCP) is used to refer to any principle that can be applied to change a determinant of behavior [27]. The term *BCP* is used interchangeably to refer to two overlapping but distinct terms, namely *Behavior Change Techniques* (BCTs) and *Behavior Change Methods* (BCMs). Taxonomies of BCTs [28] and BCMs [29] were consulted by the research team to identify BCPs suitable for changing the selected determinants of condom use. The BCM taxonomy provides methods linked to behavioral determinants. Only methods that have been “shown to be able to change one or more determinants of behavior” [29] are listed, as are the conditions or *parameters* under which they have been shown to be effective. The BCM taxonomy was used by the team to identify BCMs suitable for targeting the determinants of this intervention. The parameters of these methods were also noted.

The BCT taxonomy was initially developed to enable the coding of BCTs within existing behavioral interventions. As such, BCTs are not linked to behavioral determinants within the taxonomy. However, a recent consensus exercise has been undertaken, in which one hundred behavior change experts rated their agreement of the link between 61 BCTs, taken from the 93-item BCT taxonomy, and 26 mechanisms of action: 80% agreement was judged as consensus having been reached. This enables intervention developers to use the BCT taxonomy in a new way, that is, to select BCTs to target specific behavioral determinants. The first author (KN) participated in the consensus exercise and, accordingly, was provided with the confidential report of findings currently under preparation for publication. The findings identified BCTs to which 80% or more of the experts responded “definitely yes” when asked whether they were linked to the determinants to be targeted by the intervention. At the end of this process, a list of BCTs and BCMs identified as suitable for targeting the chosen determinants was produced.

#### Co-Design Workshop

A co-design workshop was convened to generate ideas or *practical applications* for targeting the change objectives. The intention was to create an environment in which thinking was creative, bold, and unrestricted. For this reason, the identified BCPs were set aside for this event, and attendees were encouraged to think openly about ideas that might work to target the performance objectives. Attendees included a group of 15 young people aged 16-24 years whose involvement was facilitated and supported by a sex education consultant (JH). This group was formed from the following: young people under the care of the Youth Offending Service (n=2), pupils from a city center training college (n=8), and student nurses from one university (n=5). Also in attendance were members of the steering group (KN, RC, JB, JS, and AS), a product designer, two cocreation researchers, an electronic artist, a visual artist and designer, and the head of digital for a sexual health charity.

**Figure 1:**
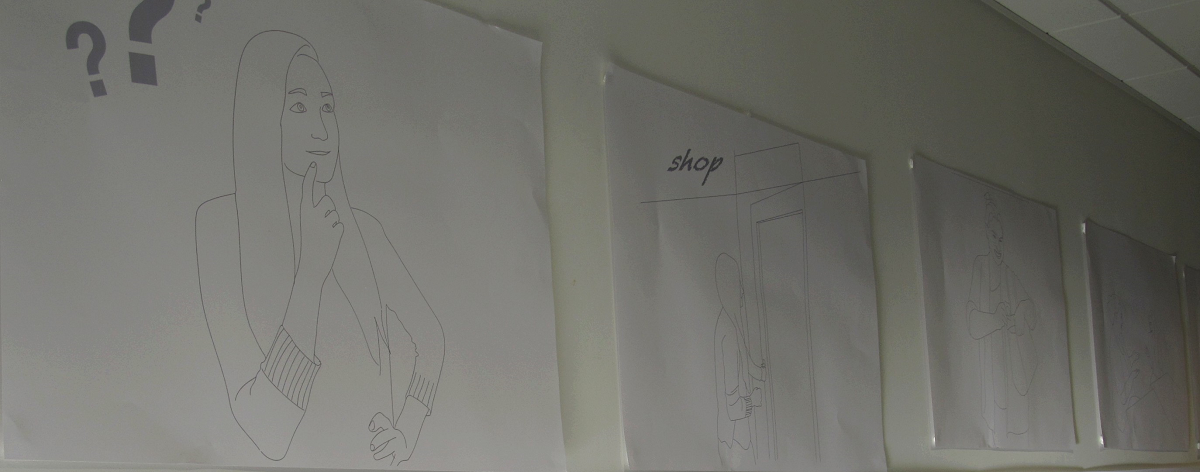
Wall display of images reflecting performance objectives.

**Figure 2:**
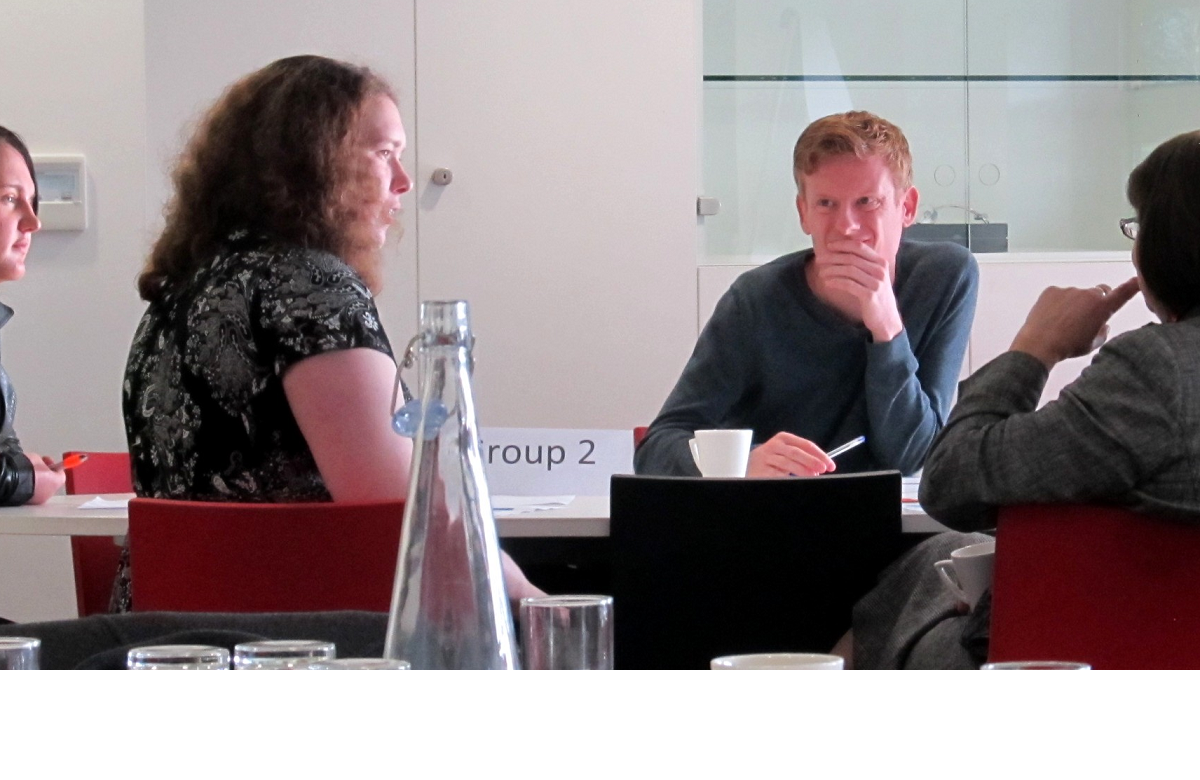
Workshop group discussion.

Ahead of the workshop, a large wall display was created with images to reflect each of the five performance objectives that were described to the attendees (see Figure 1). Attendees were split into groups so that each group was working on generating ideas for one performance objective. Each table had a variety of materials placed on it to inspire and support creativity (eg, colored pens and highlighters, paper, sticky notes, tablets, Blu Tack, and plasticine). Additionally, a description of the relevant performance objective was provided, along with questions that directed the groups to focus on the related change objectives. Everyone was encouraged to come up with novel ideas about how the intervention could address the issues presented (see Figure 2).

The workshop took place on Coventry University premises in Coventry, England, and was facilitated by KN. Throughout the day, attendees were instructed to rotate around the groups so that everyone contributed to each performance objective. This also ensured that each group had a mixture of experts in it at all times to include, as a minimum, one health psychologist, one individual with relevant clinical and public health experience, and one individual with expertise in creative techniques (eg, product designer, electronic artist, or visual artist and designer). At the start of each new rotation, groups were free to continue to work on the ideas of the previous group or to work up something new. One person who had participated in each group’s discussion always remained at the table to provide continuity.

At the end of the day, the whole group came back together and the wall display representing the five issues was populated. The whole group then discussed the ideas that had arisen and a number were further developed. After the workshop, a subgroup of the research team continued to work on and refine the ideas. Inherent BCPs were identified for each of the ideas. All ideas were then judged according to their feasibility (ie, ability to be delivered digitally and their cost), their ability to engage the priority population, and their ability to effectively achieve the change objectives. The latter was judged on the basis of whether the idea delivered an identified BCP that could be as effective in changing the relevant behavioral determinant and whether the parameters for use could be met. A final selection of ideas (ie, practical applications) was made based on these criteria.

### Step 4: Intervention Production

#### Development of Prototypes

The selected practical applications were worked up into six intervention components. The development of each component involved input from young people and experts as follows. Students from the training college (n=8) and members of the Respect Yourself Advisory Board, a group assembled to provide guidance on sexual health issues, services, and support in their area (n=6), met on multiple occasions to give feedback on prototypes for components 1 and 3. They also gave guidance on the nature of the service to be delivered through component 2. This feedback was given at face-to-face meetings; additionally, the Respect Yourself group also provided this via a private Facebook page. A youth theater group (n=25) advised on the content and design of component 6. Components 4 and 5 were informed by the same theater group who also went on to be filmed for the content.

Components 1-3 were developed with support from a product designer. A filmmaker was commissioned to film, edit, and produce the videos for components 4 and 5. Component 6 was developed in partnership with the Pleasure Project [30], an organization that campaigns for sex and relationships education that acknowledges the role that pleasure plays in sexual health decision making. Consultation with specific individuals and teams was also made with regard to the safeguarding of users of the intervention and concerning systems and processes to ensure compliance with current UK data protection legislation.

#### Website Development

The full suite of components was developed over a period of 9 months, during which time a design team worked on the branding of the intervention and on the digital architecture of a website required to host the components. The design team was appointed following an open tender process. The design team worked closely with the project lead (KN) to deliver the required functionality and look of the intervention. At a number of stages, the Respect Yourself Advisory Board provided direct input. This included, for example, commenting on intervention branding (ie, name, logo, and colors), early wireframes (ie, a set of images showing proposed functional elements of Wrapped), and copy to be used on the website. This level of user input was considered vital to ensure high levels of usability and appeal. The name for the website, Wrapped, was chosen by this group, as was the branding. Different colors and styles were uploaded onto a private Facebook page; the group discussed each of these and then nominated their favorite three. The branding with the highest number of votes was selected. See Multimedia Appendix 1 for the chosen logo.

#### Think-Aloud Study

Think-aloud interviews were conducted to test the usability of the full intervention prior to making final changes. Institutional ethical approval was received for this study and all participants were required to provide informed consent. A total of 12 young people aged 17-24 years—5 male (42%) and 7 female (58%)—were recruited through the chlamydia self-sampling website freetest.me [31]. Participants were given a £20 Amazon e-voucher in recognition of their time. Ahead of the interview, examples of intervention products (see components 1-3 in the Results section) were posted to all participants. They were instructed not to open these. Participants met virtually with a researcher via Skype for one-on-one interviews. At the start of the interview, participants were provided with the URL for Wrapped and asked to load the website. They were then asked to share their screen via Skype. This enabled synchronous viewing of the intervention by the researcher and the participant. Each participant was asked to work through the content, as they would if browsing in their own time, and to “think aloud” as they did so, verbalizing thoughts on the look, feel, content, and ease of use and navigation. Prompts were used to encourage participants to “think aloud” and to provide feedback on particular aspects. Due to the personal nature of component 6, participants were asked to view the video content in their own time and provide written feedback after the interview via email. At relevant points during the interview, participants were instructed to open the appropriate items that had been posted to them. First impressions and thoughts on whether these would be used and how they could be improved were then collected. Multimedia Appendix 2 provides the interview schedule for the think-aloud interviews. The researcher made notes throughout. Content analysis [32] was used to organize the data into themes.

## Results

### Step 1: Needs Assessment

#### Overview

The priority population for the intervention was determined to be young people aged 16-24 years. The intention from the outset was for the intervention to be embedded within the pathway of existing chlamydia self-sampling websites. These services are commissioned at a local level by public health departments under local government control and are part of the NCSP [6]. The age range that the NCSP provides chlamydia testing to is 15-24 years. The target age range for the Wrapped intervention was therefore largely determined by NCSP provision. However, the lower age limit was increased to 16 years for safeguarding reasons.

#### Evidence Review

Table 2 presents the determinants of condom use selected as the basis of the intervention as a result of the evidence review.

**Table 2:** Determinants of condom use selected for targeting based on importance, that is, the strength of association with condom use.

Determinant of condom use	Definition	Evidence regarding importance
Attitude and outcome expectancies	Extent to which people value the behavior	Overall attitude, *r*^a^=.32 [33] Affective attitude components (ie, general affect regarding condoms, for example, good or bad): adult men, *r*=.34 [34]; prospective, *r*=.37, and cross-sectional studies, *r*=.42 [35] Beliefs about pleasure, *r*=.28 [34] Beliefs about spontaneity, *r*=.35 [34] Beliefs about partner reactions, *r*=.43 [34] Belief that condoms prevent STIs^b^, including that they are reliable, will not break, etc: adult men, *r*=.24 [34]
Perceived norms	Beliefs about what is usual and acceptable	Descriptive norm, *r*=.37 [33]
Self-efficacy	Confidence in performing the behavior	Self-efficacy for condom use: cross-sectional, *r*=.24, and prospective studies, *r*=.33 [35]; correlations (cross-sectional or prospective designs included), *r*=.25 [33]
Behavioral capability^c^	Knowledge and skills required to perform the behavior	Necessary for achievement of self-efficacy
Resources	External objects and services that address barriers	Availability of condoms, *r*=.41 [33] Carrying condoms, *r*=.31 [33]

^a^The strength of the correlation, *r*, is qualified as weak (≤.1), weak to moderate (.1 to .3), moderate to strong (.3 to .5), or high (≥.5) [36].

^b^STI: sexually transmitted infection.

^c^This determinant was included on the basis that self-efficacy for condom use could not be effectively increased without first providing users with the behavioral capability to correctly apply them.

As part of the evidence review, meta-analyses examining the effect of digital interventions on the determinants of health behavior were also examined. The largest effects of digital interventions were observed for knowledge, followed by attitude and self-efficacy, albeit all were still in the small effect size range (standardized mean difference <0.5) [36]. It was clear that previous digital interventions had not been particularly effective in changing these determinants. It was not appropriate therefore for the research team to further refine the selection of behavioral determinants based on this evidence. It was acknowledged that the limited efficacy of digital interventions may in part be due to a failure as yet to establish what works to change behavioral determinants using this type of media. It was agreed that development of this intervention was therefore justified and that, in order to contribute to the knowledge base, future evaluations should seek to “unpick” the intervention in order to identify what worked or did not work to change the targeted determinants and why.

### Step 2: Intervention Outcomes and Objectives

#### Establishing Intervention Goal and Performance Objectives

The intervention goal (ie, aim) was for “young people to use condoms correctly and consistently at every instance of sexual intercourse.” The agreed upon performance objectives were as follows:

1. Decide to use condoms for vaginal or anal sex
2. Obtain condoms
  - Identify where and how to access condoms
  - Select preferred type of condom
  - Buy or request condoms
  - Maintain supply of condoms
3. Make condoms available at all times
4. Make partner aware of intention to use condoms
  - Identify when to make intention to use condoms known
  - Have plan for what one will say and do to make intention to use condoms known
  - Have plan for how to deal with, and pose solutions to, partner’s disagreement or refusal to use condoms
5. Correctly use condoms

#### Developing the Matrix of Change Objectives

Multimedia Appendix 3 displays the final matrix of change objectives.

### Step 3: Intervention Design

#### Selecting Behavior Change Principles

Multimedia Appendix 4 displays the pool of available BCPs linked to theoretical determinants of condom use.

#### Co-Design Workshop

At the end of the workshop, the wall display and a table positioned underneath were populated with numerous notes, drawings, and models to communicate the ideas discussed within the groups. Ideas ranged from simple and practical to complex and aspirational. Multimedia Appendix 5 provides a summary of the ideas generated on the day and the research team’s initial assessment of the ideas. As described in the Methods section, all ideas were judged according to their feasibility, ability to engage the priority population, and ability to effectively achieve the change objectives. A final selection of ideas (ie, practical applications) was made based on these criteria and worked up into prototypes as described in the next section.

### Step 4: Intervention Production

#### Development of Prototypes

##### Overview

The ideas generated by the workshop led to the development of six specific intervention components. These evolved and were refined through an iterative process that involved considerable input from young people and experts as described within the Methods section. The finalized components are briefly described in the following sections. Please refer to Multimedia Appendix 6 for a detailed description.

##### Component 1: Sample Pack

The sample pack is a box containing 12 condoms and two sachets of lubricant. This component aims to help young people to identify their preferred type of condom and lubricant; to help them overcome any issues around the smell, fit, and feel of condoms; and to make positive associations between condoms and pleasure (see Multimedia Appendices 7 and 8).

##### Component 2: Order Condoms

Young people are able to order condoms via a free mail-order condom distribution service. This component aims to make condoms more accessible (ie, affordable and obtainable) to young people (see Multimedia Appendix 9).

##### Component 3: Condom Carrier

The carrier is a small headphone case that has a hidden compartment for storing condoms and lubricant. This aims to increase condom availability (see Multimedia Appendix 10).

##### Component 4: Using Condoms

This video provides step-by-step instructions on how to put on a condom using a demonstrator. As well as practical tips, advice is given on how to make this part of the flow of sex. This aims to increase young people’s self-efficacy for condom use (see Multimedia Appendix 11).

##### Component 5: Discussing Condoms

This is a series of seven videos where young people talk about ways in which they have brought up condom use with partners in the past and introduced them into sex. The aim is to help users plan the best time to bring up condom use with their partner and what to do or say if they resist (see Multimedia Appendix 12).

##### Component 6: Real Life

This is a series of three videos featuring three real couples aged 18-24 years who talk about and demonstrate condom use: who does it, techniques, how to make their use part of sex. The aim is to build positive associations between condom use and pleasure (see Multimedia Appendix 13).

#### Website Development

The basis for the tailoring of intervention components were the behavioral determinants, particularly beliefs underlying these determinants that were identified as important. These were phrased as barriers to condom use; users of the intervention are asked to select which beliefs are relevant to them when they first access the website. Logic rules were written to enable allocation of relevant components to users based on their selections (see Table 3). After selecting barriers to condom use, users are presented with the website home page. This displays between one and six image and text blocks, each representing an intervention component. Users are informed that these components were selected for them based on the barriers they selected. From this point onward, users are free to browse the content, returning as frequently as they choose. While the condom sample pack and carrier were designed to be a one-off order, the condom delivery service was designed to be available to users on an ongoing basis. The video content was also made continuously available. The decision was made to make component 1 (ie, free sample box of condoms and lubricant) available to all users, that is, not to make it subject to tailoring. This was to ensure that regardless of response to tailoring statements, every user was guaranteed access to some content. It was also agreed that a sample box of condoms and lubricant was a product that all young people could benefit from and that it would provide a potentially useful incentive for encouraging first-time users to visit Wrapped.

Multimedia Appendix 14 describes the change objectives and overall behavioral determinants targeted by each component. BCTs used within each component have also been coded according to the 93-item BCT taxonomy [28].

#### Think-Aloud Study

Data were organized into five categories reflecting young people’s perceptions of intervention usability and value, as described in the following sections.

##### Website Design

Participants liked the website design, commenting that it had a “professional,” “clean,” and “friendly” look. They also commented on the tone, which they perceived as “relaxed,” “not too preachy,” and struck the right balance between sounding “fun but not too childish.”

##### Style and Tone of Language

The style of language was also appreciated: “the content is not gendered and so accessible to everyone” and “sex positive—doesn’t skirt around anything.” Views on the name Wrapped were also positive, with a number of participants commenting that is was “cryptic” and would not give away what it was about.

##### Functionality and Navigation

One feature of the original specification that was particularly unpopular was having components that “unlocked” over time. Instead, participants said that they would want all allocated components made available to them immediately. Accordingly, the developers were instructed to remove this feature.

**Table 3:** Logic rules for the allocation of intervention components to users based on their self-selected salient barriers to condom use.

Statement	Ages	Components
I can’t always get the type of condoms I want	All ages	2
I find condoms expensive to buy	All ages	2
I find buying condoms embarrassing	All ages	2
I don’t always have a condom on me when it’s needed	All ages	3
I find it awkward or difficult letting someone know that I want to use condoms	All ages	5
I’m not always able to put a condom on with confidence and ease	All ages	4
I find using condoms a turn-off	Under 18 years	N/A^a^
	18 years and over	6
I find using condoms interrupts the flow of sex	Under 18 years	4 and 5
	18 years and over	4, 5, and 6
Condoms make sex less enjoyable or pleasurable for me	Under 18 years	4
	18 years and over	4 and 6
Condoms make sex less enjoyable or pleasurable for the person I’m with	Under 18 years	4
	18 years and over	4 and 6

^a^N/A: not applicable.

##### Copy

User testing demonstrated that copy on the website, while minimal, was rarely read. All copy was therefore shortened to one-to-two sentences and additional formatting used to draw attention. In addition, while users on the whole navigated the site with ease (eg, “easy to click through” and “process feels quite classy, like a shopping experience”), the design team were instructed to make a small number of changes to improve the user experience, for example, providing clearer visual feedback to users that they had selected specific products.

##### Perceived Value of Components

Participants commented that they liked being able to personalize the condom sample pack (component 1) and condom carrier (component 3) (eg, “I like the idea of designing your own box, it’s fun”) and described the products as “discrete” and “subtle.” Participants also indicated that they appreciated and would use the products and services: “would use next to bed in drawer—perfect for bedroom,” “makes me more excited about using condoms,” “sets itself apart from other free methods of getting condoms, you know,” and “you get what you are given.”

The condom demonstration video (component 4) and the videos of young people talking about condom use (component 5) were also enjoyed: “uncensored, covers things that are often overlooked,” “interesting, relaxed and funny at times but practical advice,” “good ideas,” and “you can relate to the people in the videos.” A few people, however, found the component 5 videos too long and a bit repetitive; some also had problems with buffering. Subject to future funding, the seven videos within this section will be combined into one to address these issues.

Finally, the real-life videos (component 6) were also viewed positively: “good insight into how condoms can be a normal part of sex and not an awkward nuisance,” “nice to see sex and condoms so openly discussed and it felt really real, so easier to apply to real-life situations,” and “initially I thought they would be awkward but they were done in a classy and intimate way, which felt natural and not at all acted.” Some participants commented that they might not be to everyone’s taste but felt that their inclusion was nonetheless important: “the style was a lot more controversial than the other videos, which some people might not like, but I think it’s nice to give people that option of watching something a little more intense and serious if they want to.” Importantly, the real-life videos are only made available to users aged 18 years or older and access is preceded by a warning regarding explicit sexual content.

### Adoption and Implementation Plan

The intention is that users of chlamydia self-sampling services will be signposted directly to the Wrapped intervention immediately after making a self-sample request. All users of Wrapped will be eligible to receive component 1 (ie, free sample box of condoms and lubricant) and this will be used as an incentive to visit and register. The technical director of an STI diagnostics business worked with the research team throughout this project. Having an industry partner involved in this way was considered crucial to the success of future implementation. The technical director was on the steering group and was regularly consulted to ensure that as Wrapped continued to develop, the content and messages would be supported and that the digital architecture was compatible with their systems. Wrapped will initially be made available to users of this self-sampling website. This service fulfils approximately 150,000 self-tests per year, making potential reach high. There is also the potential to make the intervention available to other sexual health services. These include providers of other STI self-sampling websites, but also other clinical and community-based services offering STI testing. Local sexual health commissioners were also kept abreast of developments throughout the project. It is likely that any future implementation of Wrapped will require it to be commissioned at a local level. Understanding commissioning arrangements and securing the buy-in of those in a position to make funding decisions is therefore considered essential if future adoption is to be successful.

### Evaluation Plan

The study authors plan to apply for further funding to test the feasibility of trialing the Wrapped intervention. This feasibility study will consist of two parts. Firstly, a qualitative study aimed at identifying methods to enhance the recruitment and retention of participants. This will be followed by a feasibility randomized controlled trial (RCT) to examine the success of these methods. Accordingly, the primary outcome measures will be the rate of recruitment and attrition. If such a feasibility study demonstrates that it is possible to recruit and retain sufficient participants, then a full RCT will be conducted to examine the effect of the intervention on the primary outcome, namely STI incidence.

## Discussion

### Principal Findings

This paper describes the development of Wrapped, a fully automated, tailored, digital behavior change intervention for young people aged 16-24 years accessing chlamydia self-sampling websites. Wrapped aims to reduce the incidence of STIs among this group by increasing correct and consistent condom use with sexual partners. Wrapped was developed using Intervention Mapping [22]. This provides a robust framework for the development of interventions grounded in theory and evidence. In accordance with calls for full disclosure of intervention development and content [37], this paper provides a transparent and detailed account of this process and, using supplementary files, a full manualization in line with the template for intervention description and replication (TIDieR) guidance [38].

Key determinants identified as important and targeted by the intervention are attitude toward condoms, perceived norms for condom use, self-efficacy for condom communication and use, behavioral capability for condom use, and resources to increase condom accessibility. The intervention addresses these determinants through the following six different components: a condom sample pack, condom postal distribution service, a condom carrier, a condom demonstration video, a series of videos on communication about condom use, and erotic films of real couples discussing and demonstrating condom use. Each component operates in isolation and users are allocated these components when they first access the intervention in accordance with their self-identified salient barriers to condom use.

The usability of Wrapped was examined using a think-aloud study. Participants reported that Wrapped had high levels of design and was intuitive to use. They liked the branding and graphics, which they reported had a stylish and friendly feel. The importance of developing engaging digital interventions should not be underestimated. Consumers of digital media, especially young people, have come to expect quality products that are enjoyable to use and easily navigable. A great deal of care and attention was put into these aspects by the research team. A substantial budget was set aside for development of the digital interface, and the design team was selected through open competition. Furthermore, the look, feel, and functionality of Wrapped was guided throughout by a working group of young people. The tone of copy used throughout the site and on materials accompanying products was also carefully considered. In line with advice received from the working group, all copy has a sex-positive tone and is gender neutral and no assumptions are made about sexual identity. The care taken to get the tone right was worthwhile as it was frequently commented on by participants in the think-aloud study. Participants also reported that they liked being able to select personalized products; the condom carrier comes in four colors and has color-matched headphones, and the condom sample pack had four variations of box to choose from along with four different insert designs, creating 16 possible combinations. It is hoped that this level of personalization will encourage users to order products and increase users’ sense of ownership and use.

### Comparison With Prior Work

As far as the authors are aware, this is the first digital sexual health intervention to tailor content to behavioral determinants. It is proposed that the tailoring of content will enhance users’ active engagement with the intervention and potentially enhance consolidation of learning and behavior change due to relevance of the content to the user’s own situation. Wrapped is also one of the few sexual health interventions to use the eroticization of condoms as a strategy for achieving behavior change [39], specifically through videos of real couples discussing and demonstrating condom use (component 6). This approach has been found to lead to more risk-preventive sexual attitudes and behaviors [40]. Interventions embedding such strategies are rare, perhaps due to real or perceived resistance from stakeholders such as parents, religious groups, or sexual health commissioners. However, participants in the user testing responded positively to these videos. Wrapped includes an online condom distribution scheme, which is made available to users who indicate that they experience difficulties obtaining condoms. This is in line with recent guidance issued by the National Institute for Health and Care Excellence, that condom distribution schemes providing a choice of free condoms and lubricant be made easily available to young people at risk of STIs [41].

### Strengths and Limitations

Intervention Mapping proved to be a valuable tool to guide development of the intervention. It provides a robust and transparent process that results in an intervention that is not only grounded in theory and evidence, but also in the needs of the intended recipients. Furthermore, involvement of stakeholders, particularly in this case, including sexual health doctors, sexual health commissioners, and an online STI self-sampling service, increases the likelihood that the resulting intervention will be acceptable to the organizations required to adopt it.

One limitation of the approach taken in this study relates to the needs-assessment stage. Users of Intervention Mapping are advised to draw upon multiple sources of evidence, such as literature reviews and qualitative and quantitative research, to identify determinants to be targeted by the intervention. Resource limitations meant that the needs assessment for Wrapped was restricted to examination of evidence from a review of reviews. While this is a robust approach to identify high-level determinants, because only those with at least a small-to-medium effect on condom use are taken forward, it should be acknowledged that this fails to identify more nuanced but potentially important beliefs held by the target population. These may relate, for example, to their age or risk status. This more-detailed insight would have been helpful at the point of identifying change objectives, for example, understanding any particular outcome expectancies influencing negative attitudes toward condoms among this group that should be targeted by the intervention.

The most challenging aspect of development was related to step 3, intervention design. As part of this stage, BCMs suitable for targeting the behavioral determinants were selected as per the guidance. Tables of BCMs capable of achieving change in behavioral determinants are provided to facilitate this process [29]. Another dominant framework that exists to guide intervention development is the Behavior Change Wheel [42]. The equivalent to BCMs in this context are BCTs. There are no such tables for the selection of BCTs, but recent work to establish expert consensus on the link between BCTs and behavioral determinants has enabled tentative conclusions to be made. In this project, the team drew on both taxonomies to select relevant BCPs: a term used to cover both BCMs and BCTs. Due to the prominence of the BCT taxonomy, in the United Kingdom at least, the research team wanted to use this to support selection of BCPs for Wrapped. On reflection, however, this did not enhance development and simply added an additional layer of unnecessary complexity. Given the explicit links between BCMs and behavioral determinants afforded by Intervention Mapping, the authors would advise others using the Intervention Mapping framework to select BCMs alone. However, there may be merit in coding finished interventions according to the BCT taxonomy, as was also done in this case, given that this enables direct comparison with other behavior change interventions coded in the same way. This can be useful, for example, when synthesizing existing evidence that aims to draw conclusions about which BCTs work best to target particular behaviors, determinants, or populations.

### Conclusions

As demonstrated in this article, Wrapped was developed to ensure that not only was it theory-based and grounded in the needs of the target audience, but also had high levels of design and appeal. Whether it is an effective intervention for increasing condom use among users of STI self-sampling websites will be tested in a future trial. If the results are positive, Wrapped will have good potential reach as it can be easily embedded within STI self-sampling websites, which are becoming increasingly popular [7]. With the increased digitalization of health services in an effort to reduce costs, this could be an important means of providing sexual health promotion and support to young people who would otherwise receive these face-to-face.

## Appendix

Multimedia Appendix 1 Wrapped logo.

Multimedia Appendix 2 Think-aloud interview schedule.

Multimedia Appendix 3 Finalized matrix of change objectives.

Multimedia Appendix 4 Pool of available Behavior Change Principles (BCPs) linked to theoretical determinants.

Multimedia Appendix 5 Summary of ideas for intervention components resulting from the co-design workshop.

Multimedia Appendix 6 Detailed description of intervention components.

Multimedia Appendix 7 Image of Wrapped webpage showing options for component 1 (Sample Pack).

Multimedia Appendix 8 A page from the booklet contained within component 1 (Sample Pack).

Multimedia Appendix 9 Selection of condoms available via component 2 (Order Condoms).

Multimedia Appendix 10 Image of component 3 (Condom Carrier, in choice of four colors).

Multimedia Appendix 11 A selection of stills taken from component 4 (Using Condoms).

Multimedia Appendix 12 One of the seven videos making up component 5 (Discussing Condoms).

Multimedia Appendix 13 Excerpts from the videos making up component 6 (Real Life).

Multimedia Appendix 14 Specification of the Behavior Change Techniques (BCTs), targeted determinants, and change objectives.

## Abbreviations

BCM: Behavior Change Method
BCP: Behavior Change Principle
BCT: Behavior Change Technique
NCSP: National Chlamydia Screening Programme
RCT: randomized controlled trial
STI: sexually transmitted infection
TIDieR: template for intervention description and replication
