# Supplementary figures and images for: An Intervention to Increase Condom Use Among Users of Chlamydia Self-Sampling Websites (Wrapped): Intervention Mapping and Think-Aloud Study
Source: JMIR Form Res. 2019 May 1;3(2):e11242. doi: 10.2196/11242 (PMC6658247; doi:10.2196/11242)

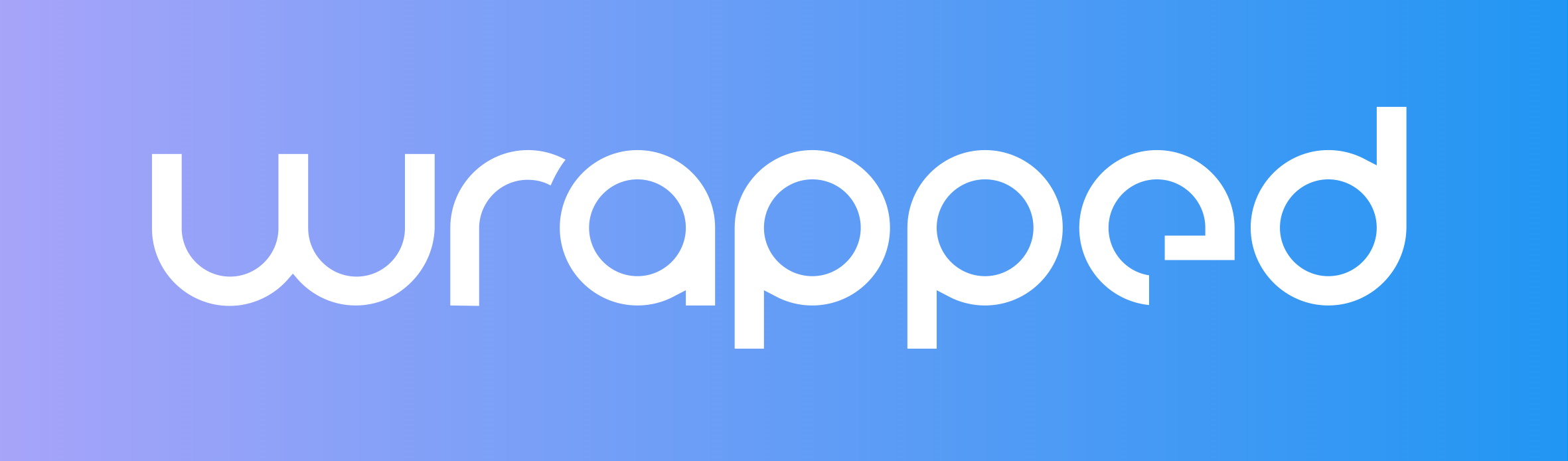

Supplement: Multimedia Appendix 1 [file formative_v3i2e11242_app1.png]

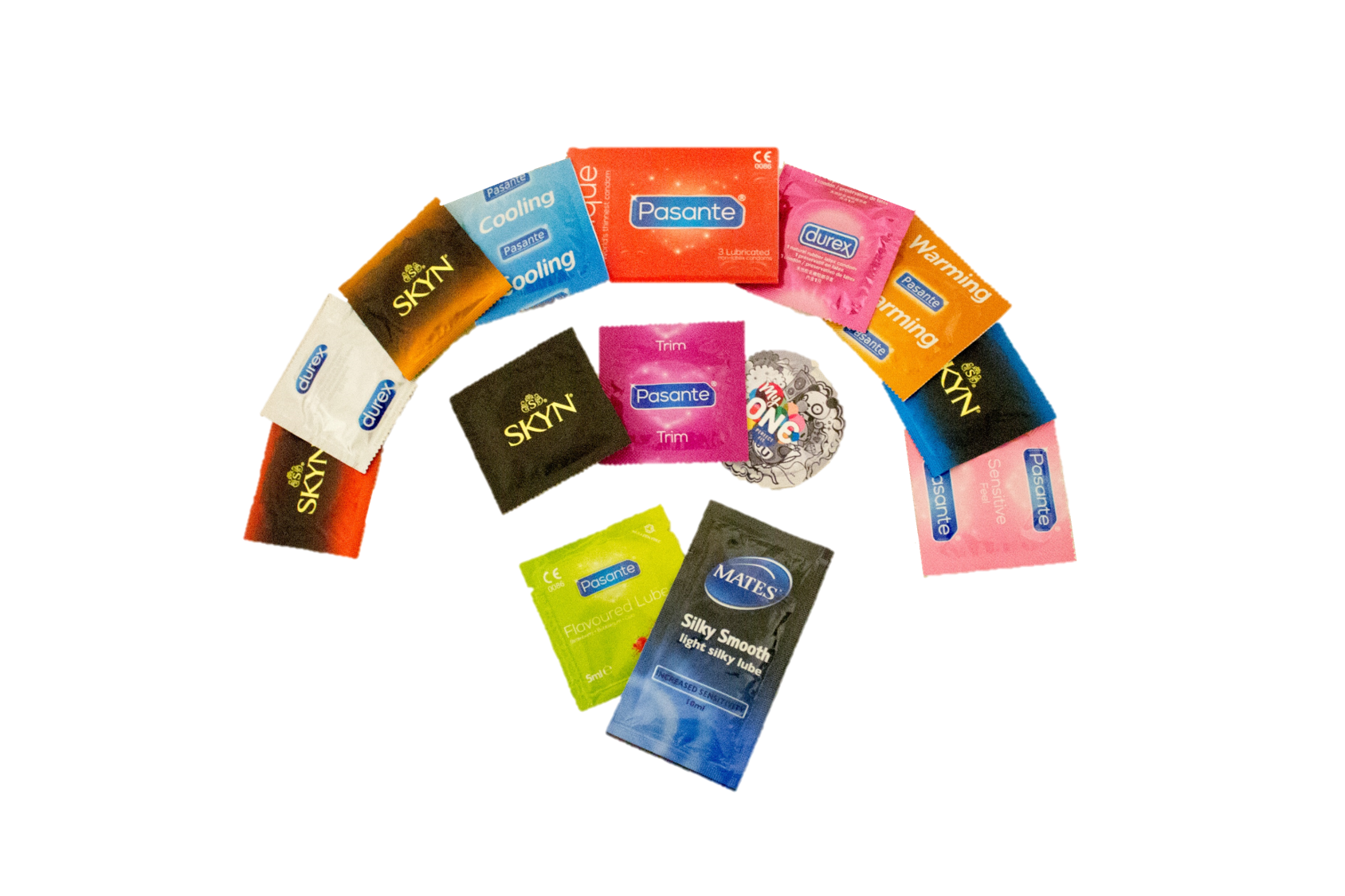

Supplement: Multimedia Appendix 9 [file formative_v3i2e11242_app9.png]

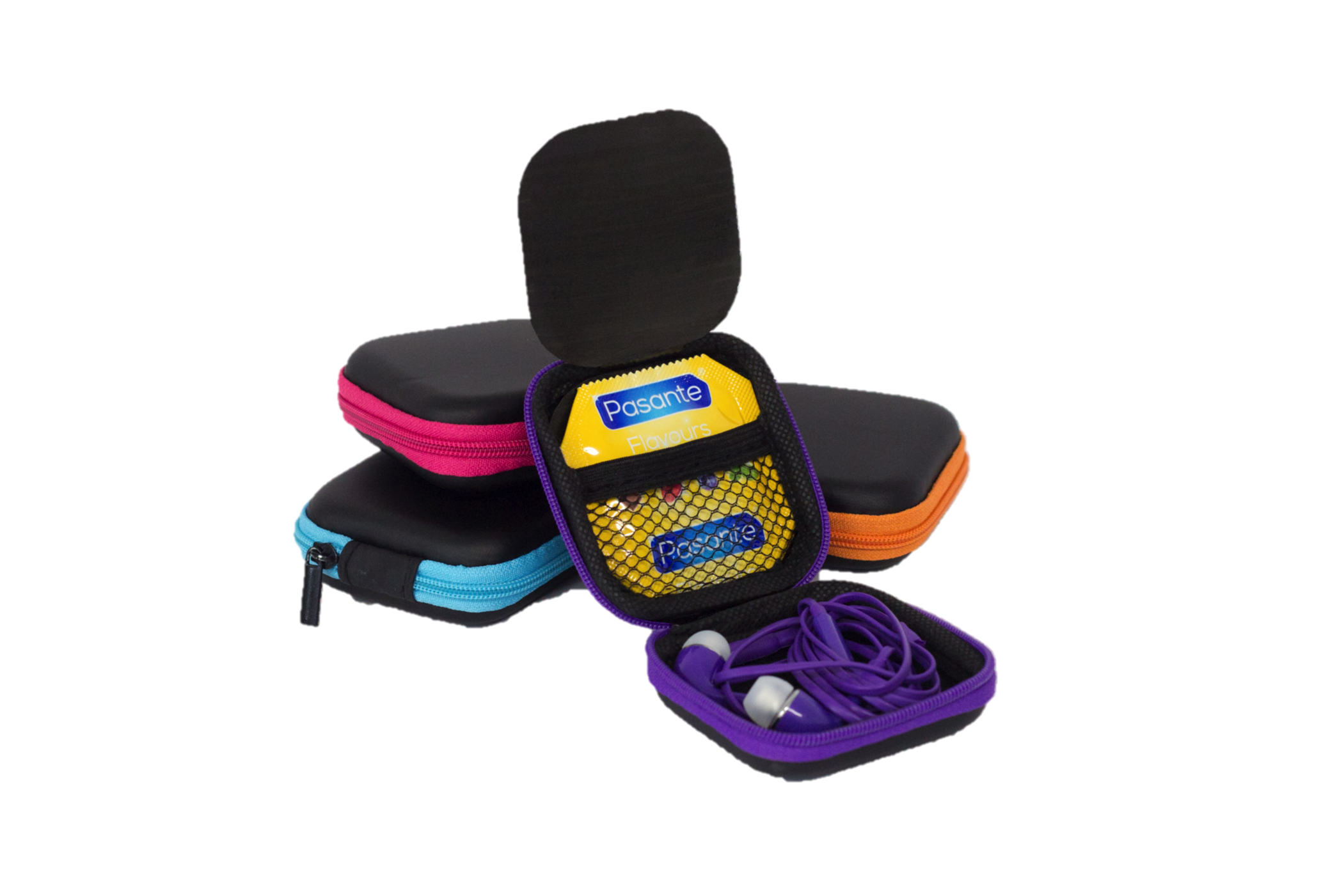

Supplement: Multimedia Appendix 10 [file formative_v3i2e11242_app10.png]

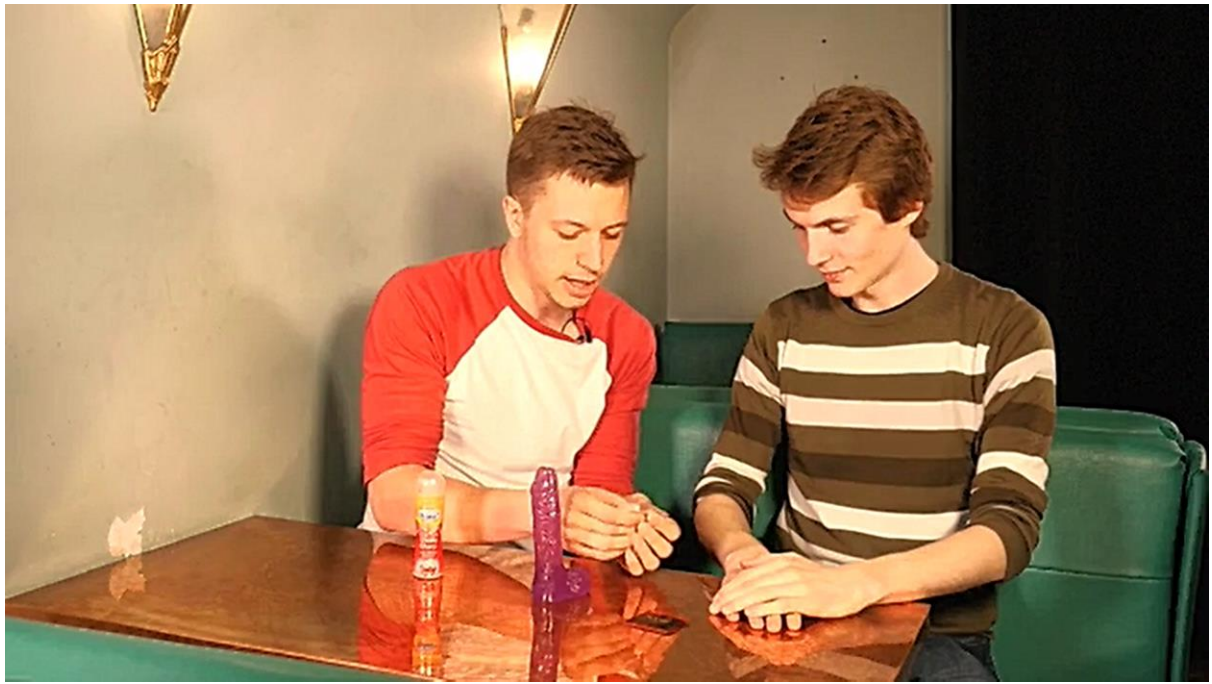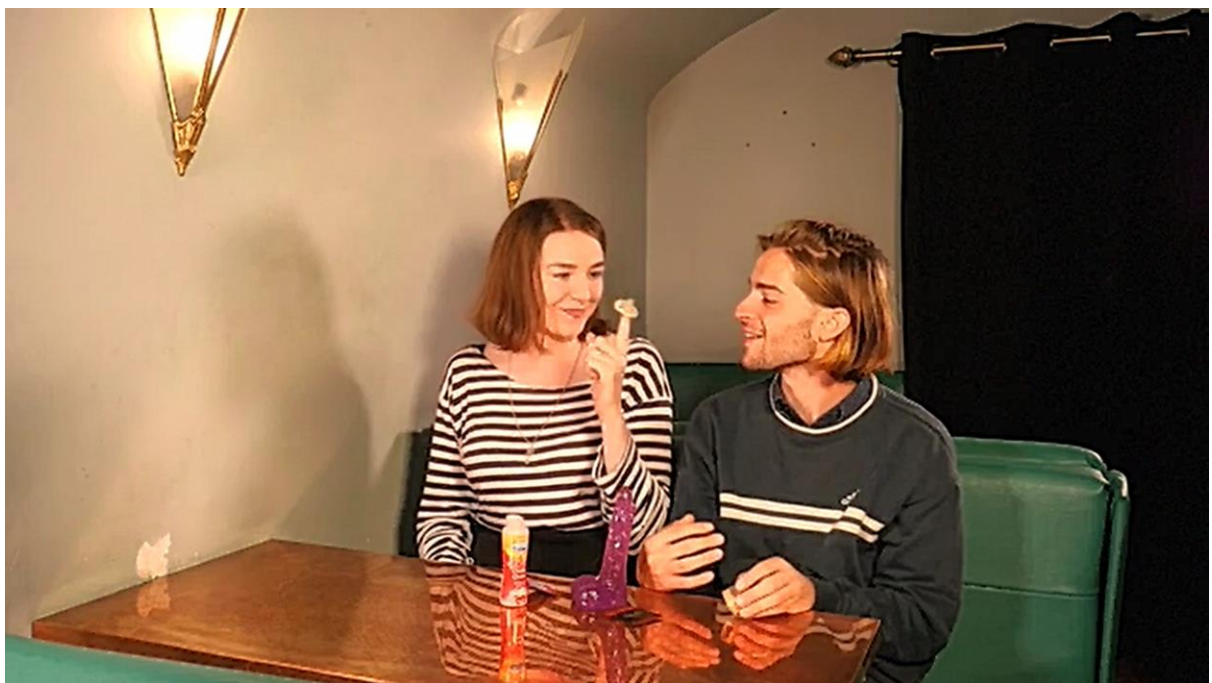

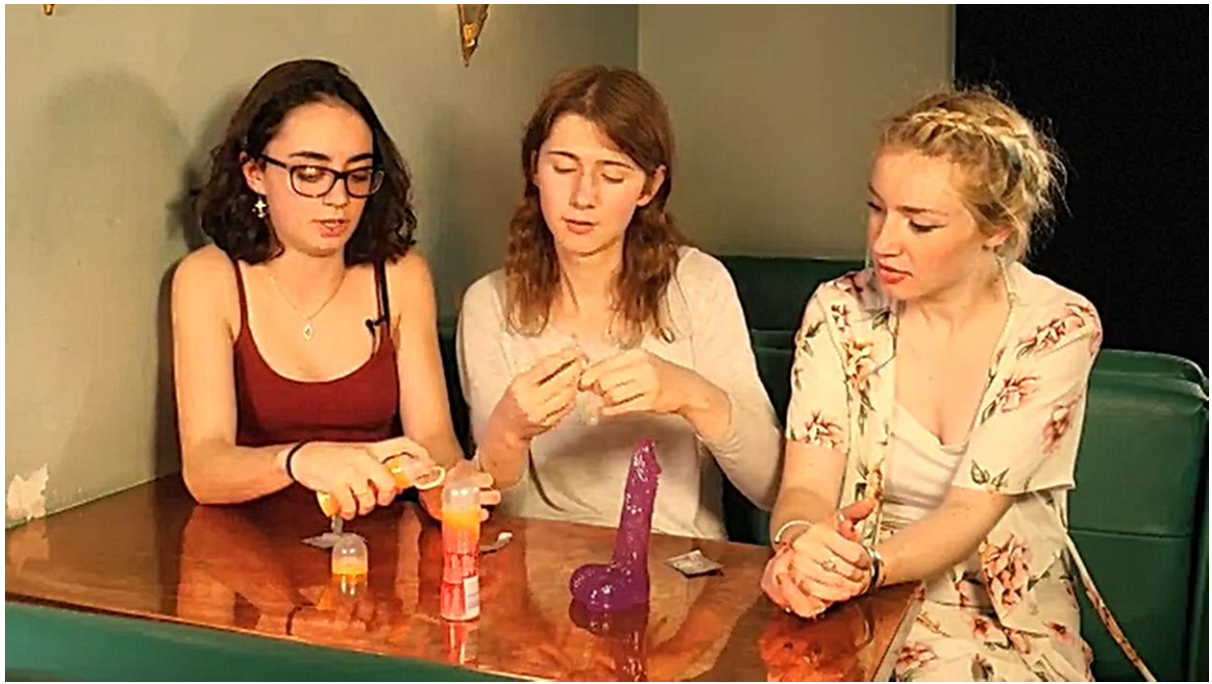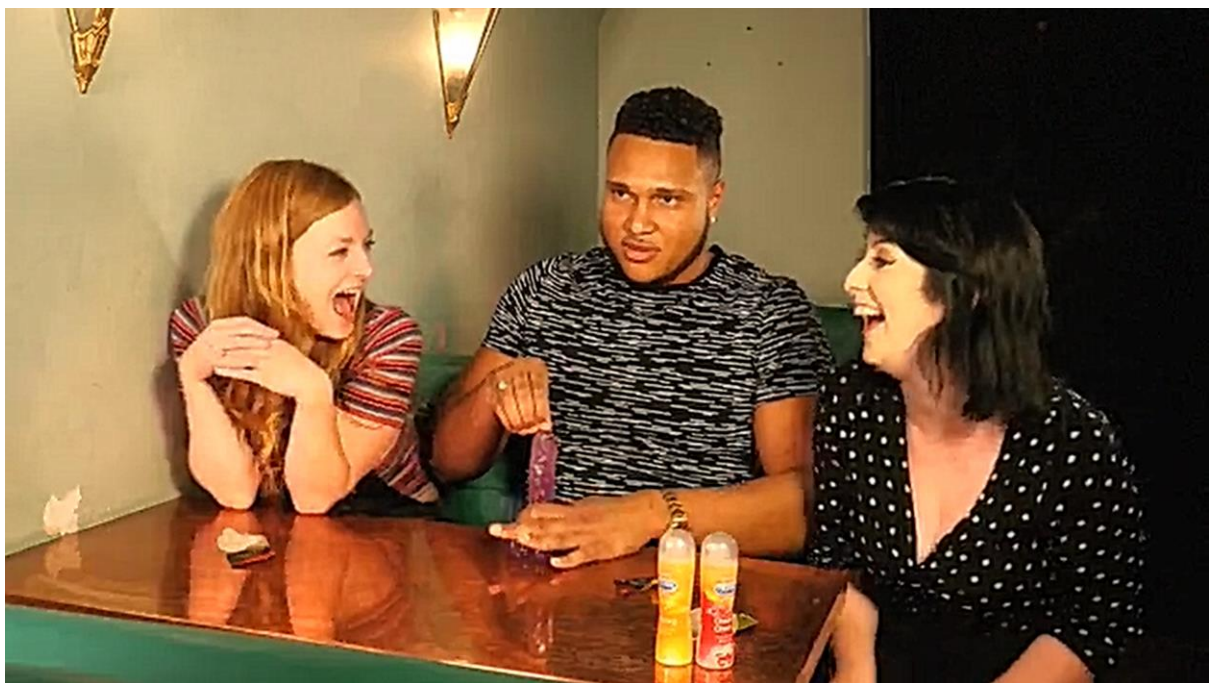

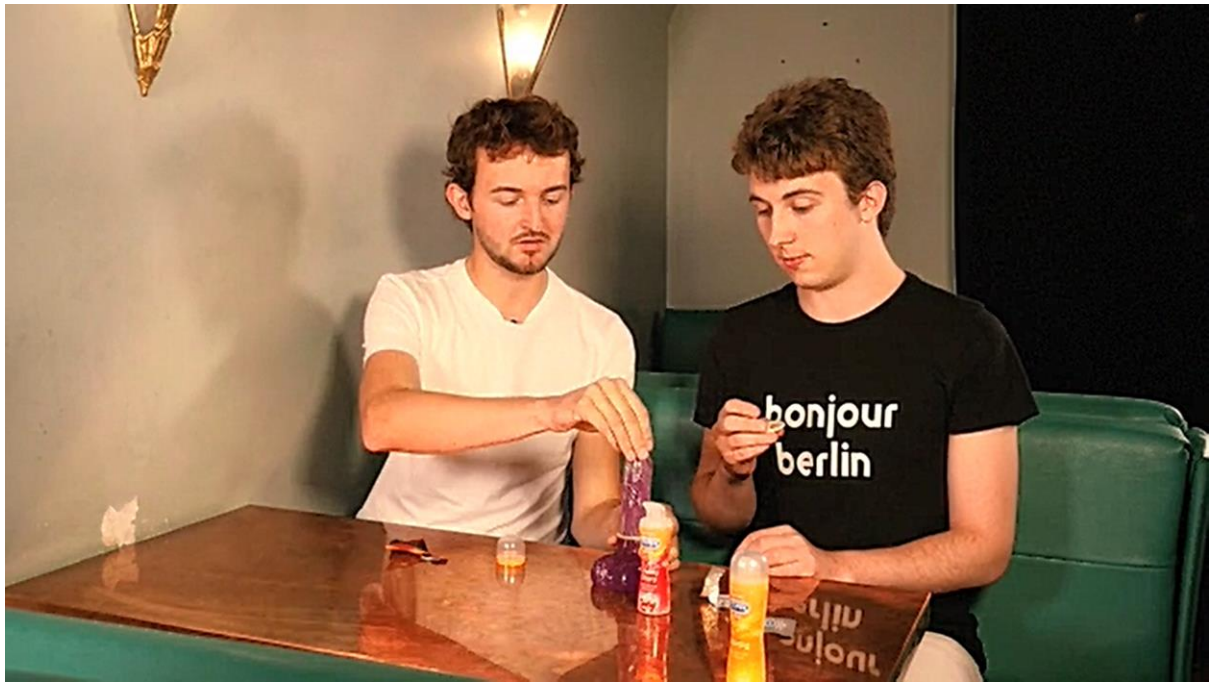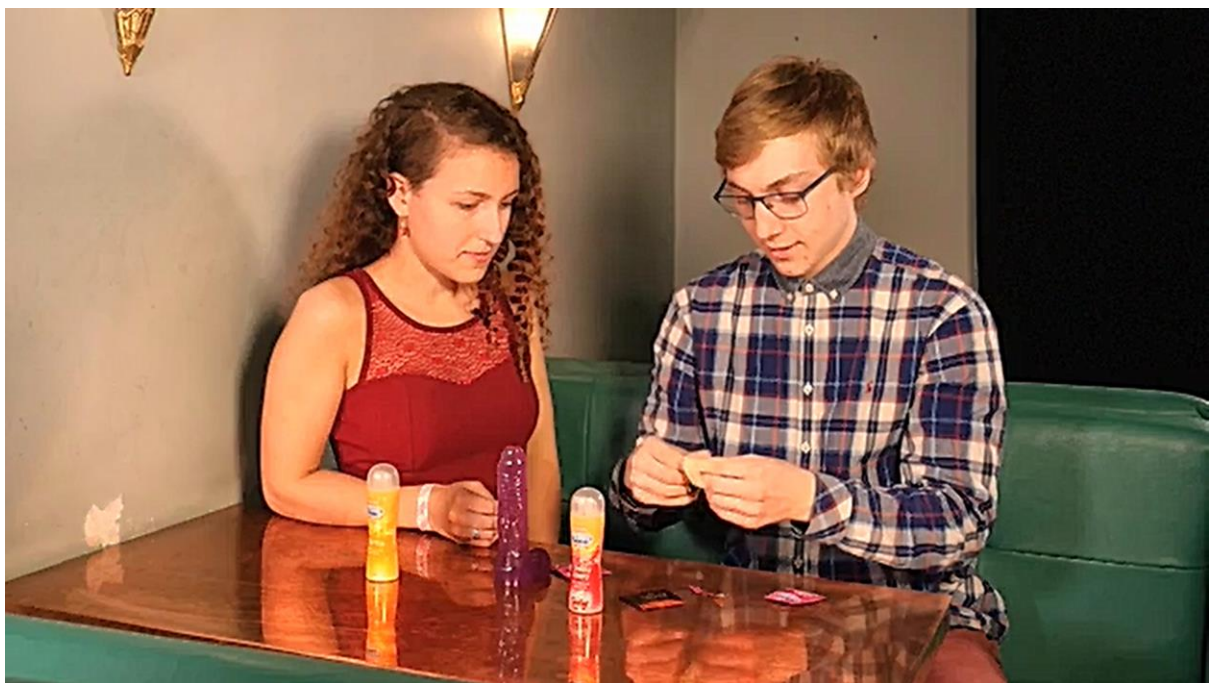

Supplement: Multimedia Appendix 11 [file formative_v3i2e11242_app11.pdf]
