# Supplementary material for: An Intervention to Increase Condom Use Among Users of Chlamydia Self-Sampling Websites (Wrapped): Intervention Mapping and Think-Aloud Study
Source: JMIR Form Res. 2019 May 1;3(2):e11242. doi: 10.2196/11242 (PMC6658247; doi:10.2196/11242)
Supplement: Multimedia Appendix 2 [file formative_v3i2e11242_app2.pdf]

Multimedia appendix 2: Think aloud interview schedule

Notes for researchers (START)

- Post sample box of condoms and condom carrier ahead of meeting (NB these are for them to keep)
- If meeting via Skype, make sure send invite to connect with participant ahead of meeting
- Send them link to Wrapped ahead of meeting
- Go through PIS. Give opportunity to ask questions and check happy to proceed
- You must be sensitive to needs of the participant throughout the session. If you detect any sign of upset or discomfort ask them if they wish to have a break or to terminate the session. Remind participant that they can choose not to view any sections of Wrapped that they are not comfortable with
- If participant allocated to the condom demo – briefly explain what it is first and check that they are happy to view
- If participant allocated to explicit videos, then they are to watch after session (notes below)
- It would be a good idea to have participants who test on a range of devices PC, laptop, phone (apple and android)
- Ensure that the version of Wrapped given to each participant is appropriate for their age (<18 years, or 18+)

Wrapped Think Aloud Study - Schedule

- Ask the participant to log-on to Wrapped
- Ask them to complete the statements section and then to work through the resource
- Encourage them to say out loud what they are thinking at as they look through each page
- Ask them to have a go at ordering any products that they are allocated to
- Ask them to open samples posted to them (NB the carrier may not be relevant) and ask for feedback
    - First impressions
    - Would you use this?
    - How could we improve this?

NB if the user is aged 18 or over they may be allocated component 6 (videos with adult content). If this is the case, ask them to view once the session has ended and to email their feedback to you. Send them email with questions for them to respond to.

Once worked through entire resource ask:

- Do you like the look of the website?
- Could the presentation of information be improved?
- Is it easy to use?
- Can you find your way around?
- Is it enjoyable to use?
- Do you feel you can trust the information provided?
- Do you find the information valuable?
- Would you use Wrapped?
- Would you recommend it to others? Why/why not?
- Instead of viewing all content at once as you are today, users will be able to view one new component each day as they become ‘unlocked’. What would you prefer – to receive all content straight away, or over a period of days?
- Currently, emails are used to communicate with users e.g. to alert them to new unlocked content. Would you prefer text messages? Or WhatsApp?
- What do you think of the choice of inserts for the condom sample box? Is there one that you would be happy to choose?
- Currently, there is only the option to have products sent to a home address. Would you like to be able to pick up an item from a depot e.g. Collect+, Amazon locker, Doddle?
- What do you think of the videos? Which is your least favorite (why)? Do the young people in the videos represent you and your friends? What do you think about the length of the videos?
- You accessed the site directly from a link that we provided but ordinarily, users will be invited to visit the site following their request for an STI test. The invite reads as follows:
  Would you like a free sample pack of twelve condoms? Register with our sister site Wrapped to receive this and see whether you are eligible for other free products and services

What do you think about this? Would you click on the link? How could it be improved?

Finally….

- The purpose of this resource is to support young people to use condoms.
  - Would you find it useful/helpful?
  - Has it changed the way you think about condoms at all?
  - Do you think that it might change how likely young people are to use condoms? In what way? Would it work for you?

Notes for researcher (END)

- Debrief participants at the end of the session and refer them to the RespectYourself.info website for further information, support and access to services.
- Ask if they would be happy to be contacted about taking part in future Wrapped working group (if so record this)
- Send them the email requesting feedback on component 6 (if relevant)
- Send them the £20 amazon e-voucher
