# Supplementary material for: An Intervention to Increase Condom Use Among Users of Chlamydia Self-Sampling Websites (Wrapped): Intervention Mapping and Think-Aloud Study
Source: JMIR Form Res. 2019 May 1;3(2):e11242. doi: 10.2196/11242 (PMC6658247; doi:10.2196/11242)
Supplement: Multimedia Appendix 3 [file formative_v3i2e11242_app3.pdf]

Multimedia appendix 3: Finalised matrix of change objectives

	**Personal**				**External**
**Performance Objectives**	**Attitude**	**Perceived Norms**	**Self-efficacy**	**Behavioural Capability**	**Resources**
**1. Decide to use condoms**					
	Express belief that condom use doesn't have to reduce own sexual pleasure				
	Express belief that condom use won't reduce partner's sexual pleasure				
	Express belief that sex can be enjoyable with condoms				
	Express belief that condom use won't reduce spontaneity				
	Express belief that condoms are effective and reliable (won't break, slip etc) in preventing STIs				
		Recognise that others in their peer			

		group use condoms			
**2. Obtain condoms**					
**2.1 Identify where and how to access condoms**				Identify where and how can access	Identify or develop a service to enable easy access to condoms
				Plan where and how to access	
**2.2 Select preferred type of condom**				Identify preferred type of condom	Identify or develop service that provides a choice of condoms
**2.3 Buy/request condoms**			Express belief that able to buy/request		
**2.4 Maintain supply of condoms**				Monitor condom supply and obtain more before running out	
**3. Make condoms available at all times**					Identify a place to store supply of condoms
					Identify place to carry condom (that will ensure always available)
				Replace used condom asap after use	
**4. Make partner aware of intention to use condoms**				Describe the best time(s) to make partner aware of intention	

**4.1 Identify when to make intention known**				Identify cues that indicate latest point at which could communicate intention	
**4.2 Have plan for what will say/do to make intention known**			Express belief that can communicate intention	Describe what will say and/or do to communicate intention	
**4.3 Have plan for how to deal with/ pose solutions to partner's disagreement/ refusal to use condoms**			Express belief that can persist with communication in the face of resistance (and be prepared to not have sex)	Describe what will say/or do if partner disagrees/refuses	
**5. Correctly use condoms**					
			Express belief that can correctly apply and remove	Demonstrate that can correctly apply and remove	
