# Supplementary material for: An Intervention to Increase Condom Use Among Users of Chlamydia Self-Sampling Websites (Wrapped): Intervention Mapping and Think-Aloud Study
Source: JMIR Form Res. 2019 May 1;3(2):e11242. doi: 10.2196/11242 (PMC6658247; doi:10.2196/11242)
Supplement: Multimedia Appendix 4 [file formative_v3i2e11242_app4.pdf]

Multimedia appendix 4: Pool of available Behaviour Change Principles (BCPs) linked to theoretical determinants of condom use

**Determinants of condom use**	**Behaviour Change Principles (BCPs)**	
	**Behaviour Change Techniques[a]**	**Behaviour Change Methods**
Attitude towards condoms / outcome expectancies	Information about health consequences<br><br>Information about emotional consequences<br><br>Information about social and environmental consequences<br><br>Salience of consequences<br><br>Pros and cons<br><br>Framing/re-framing<br><br>Comparative imagining of future outcomes<br><br>Incentive (outcome)<br><br>Reward (outcome)	Classical conditioning<br><br>Self-re-evaluation<br><br>Environmental re-evaluation<br><br>Shifting perspective<br><br>Arguments<br><br>Direct experience<br><br>Elaboration<br><br>Anticipated regret<br><br>Repeated exposure<br><br>Cultural similarity
Perceived norms for condom use	Information about others' approval<br><br>Social comparison	Information about others' approval<br><br>Resistance to social pressure<br><br>Shifting focus<br><br>Mobilising social support

		Provide opportunities for social comparison
Self-efficacy for condom use (accessing, carrying, negotiating, using)	Set graded tasks<br><br>Behavioural practice/rehearsal<br><br>Verbal persuasion about capability<br><br>Problem solving	Guided practice<br><br>Enactive mastery experiences<br><br>Verbal persuasion<br><br>Improving physical and emotional states<br><br>Reattribution training<br><br>Self-monitoring of behaviour<br><br>Provide contingent rewards<br><br>Cue altering<br><br>Public commitment<br><br>Goal setting<br><br>Set graded tasks<br><br>Planning coping responses
Behavioural capability (accessing, carrying, negotiating, using)	Graded tasks<br><br>Behavioural practice rehearsal<br><br>Instruction on how to perform the behaviour<br><br>Habit reversal	As above (self-efficacy)

Resources (accessing & carrying)	Restructuring the physical environment<br><br>Adding objects to the environment<br><br>Avoidance/Reducing Exposure to Cues for the Behaviour<br><br>Prompts/cues<br><br>Remove aversive stimulus<br><br>Restructuring the social environment<br><br>Social support practical	Systems change<br><br>Participatory problem solving<br><br>Coercion<br><br>Advocacy and lobbying<br><br>Modelling<br><br>Technical assistance

[a]BCTs where the proportion of experts rating ‘definitely yes’ is 0.8 or above in expert consensus exercise
