# Supplementary material for: An Intervention to Increase Condom Use Among Users of Chlamydia Self-Sampling Websites (Wrapped): Intervention Mapping and Think-Aloud Study
Source: JMIR Form Res. 2019 May 1;3(2):e11242. doi: 10.2196/11242 (PMC6658247; doi:10.2196/11242)
Supplement: Multimedia Appendix 5 [file formative_v3i2e11242_app5.pdf]

Multimedia appendix 5: Summary of ideas for intervention components resulting from the co-design workshop

Decision to use condoms

1) Buddy – at key steps through the testing process, the user receives messages from a virtual 'buddy'. These could be videos, text messages, other. The buddy is also going through testing and offers support, empathy plus BC messages.

Or….

2) Machine Learning/AI – synchronous communication between the user and virtual 'something' (could be the test pack itself). It responds to users input; BC messages provided; Initially a real person will need to respond but then AI will build up back of suitable responses so that can work on own. We could use beacons in testing packs to communicate with users' phone. Could also be more generally used for providing advice to young people around sex and relationships.

Or….

3) Pack of different types of condoms to try out and choose favourites?

Purchase/obtain condoms

Develop a service providing free condoms to users. Key features:

- Get selection pack for first order so can try out different types/sizes
- Set up standing order for future delivery (type and timing); or text message sent and user to confirm need
- Simple ordering system
- De-medicalised : present condoms as a consumer product not a safety/health product
- Could have membership levels: basic, silver, gold. Perks? E.g. could offer users opportunity to upload any image to be printed on condom wrapper (NB would need peel of sticker indicating code to reassure that correct type). This could be gold level. The different membership levels would be earned through contribution to content e.g. condom reviews, video/text uploads sharing experiences of negotiating condom use etc. or by recommending service to a friend(s)
- Condoms presented in DVD style presentation case with favourites displayed (like 'Graze'). Cardboard sleeve over the top which could be replaced with a cardboard DVD sleeve
- Packaging must be neutral/discrete
- Could be delivered to home address or use Collect Plus service (there is a database of sites that we can get access to)

- Animation/video demo re how to apply condom – separate for males/females
- Need to be careful not to masculinise service – market towards women too
- Include testimonies from men and women re their experience of using condoms
- Consider partnering up with TheyFit, or maybe a more well know brand such as Durex or Pasante
- Near field communication – tap DVD case with phone to bring up web app to order more condoms

Carrying condoms

The DVD case can be used to store condom supply. Some people will feel comfortable carrying condoms, and others less so and will want them hidden. Options we could develop for discrete carrying include developing something to fit in with:

Keys – fob for key ring that could double up as torch, or to hold photo

Phone

Wallet/purse

NB to produce nice product will need buy-in from commercial partners – how can we incentivise

Geolocation – consider using beacons that phone could pick up on to remind user to take condom out with them if leaving house in evening. Similarly could put them in venues (bars/clubs) to remind use and/or in hotspots to indicate to user that can obtain condoms from nearby location – could use tokens for that.

App could link to facebook events, Siri/Cortana etc & cue ordering of condoms or reminders to take

Negotiation

- Create short film with clear message which relates condom use to everyday negotiation activity (like the 'cup of tea' film) e.g. negotiating what film to see at cinema
- Get behind Durex campaign to create condom emoji
- Campaign to empower women to carry condoms and request/negotiate condom use (along the lines of 'that girl can' (sport England)
- Using VR to create different scenarios that respond to and play out ending – could put google cardboard in testing packs or condom packs
- Show testimonies of others talking about condom use
- Encourage users to make plan for when will introduce condom use

- Give message that sex can be more pleasurable if not having to think about this negotiation – ge this out the way early on
- You pee, I pee (joint testing pack for when stop use); plus guidance on when to decide when is the time to stop using

Pleasure

Link to positive porn or feminist porn website(s) e.g. Make Love not Porn showing couples having sex using condoms – could show communication about condoms, putting on condoms (by male/female), could also work by building up positive associations of sex with condoms amongst users.

Existing intervention being developed by University of Southampton – digital version of brief clinic based intervention in which condom demo provided and then user instructed to test out (masturbate with) different condoms at home until find favourite, plus when trying out instructed to focus on positive sensations. This could work well alongside the initial selection pack of condoms sent out.
