# Supplementary material for: An Intervention to Increase Condom Use Among Users of Chlamydia Self-Sampling Websites (Wrapped): Intervention Mapping and Think-Aloud Study
Source: JMIR Form Res. 2019 May 1;3(2):e11242. doi: 10.2196/11242 (PMC6658247; doi:10.2196/11242)
Supplement: Multimedia Appendix 6 [file formative_v3i2e11242_app6.pdf]

Multimedia appendix 6: Detailed description of intervention components

Component one: Sample Pack – discover your favourite condom

See multimedia appendix 6 and 7.

This is a box containing twelve types of condoms (different brands, sizes, textures, thicknesses), and two types of lube for users to try out. It is delivered to users in discrete packaging (image of packaging shown to users). The box includes step-by-step instructions on how to correctly apply condoms. The aims of this component are to help young people to identify their preferred type of condom/lube, to help them overcome any issues around the smell, fit & feel of condoms, and to make positive associations between condoms and pleasure by instructing users to play around with them on their own i.e. getting them out of packet, feeling them, unwrapping, smelling, tasting etc. Users will be encouraged to put the condoms on themselves, or insert in to their vagina using fingers/dildo, and if they feel comfortable, to go on to masturbate. It will be suggested that users try out all of the different types of condom to identify their preferred type(s). Users will also be encouraged to practice putting condoms on until can do so confidently and with ease (suggest that those without a penis use a dildo or other substitute e.g. banana).

The box itself is designed to be a permanent store for condoms at home. The bottom of the box has the message ‘running low? Get some more in’. This is a cue to remind users to replenish their supply. Users are able to select from four different designs for both the exterior and interior of the box giving a total of 16 possible combinations.

Component two: Order Condoms – order free condoms

See multimedia appendix 8.

Users can register with a service that will deliver their preferred condoms to a chosen address (in discrete packaging – image of packaging shown to users). Users will be able to select and change their preferred type(s) of condom and delivery address. Condoms will be free. Delivery will be ad hoc and triggered by an online order from the user. Each user will be limited to one order per month. Each order consists of one type of condom in bundles of six or twelve. Users can also add three packets of lube to their order. A reminder email will be sent to users when they are able to order more condoms. As well as offering this service, users will be linked to information about off-line places where they can access condoms (both online through hyperlinks to services and through written information provided with each delivery).

Users will be able to post reviews of condoms/lube within the order condoms area of Wrapped.

Component three: Condom Carrier – stash your stuff in this handy free case

See multimedia appendix 9.

A product for discretely carrying condoms when out and about, delivered to users in discrete packaging (image of packaging shown to users). This is a small headphone case with a discrete, hidden compartment for up to two condoms, or one condom and a sachet of lube. Behind the condom is the message ‘Replace me!’ to act as a cue to replace the condom after use. The case is not intended to be the sole option for carrying condoms but rather to be used as one of a number. Messaging within Wrapped when ordering this carrier, and also inside the carrier when received, encourages users to place condoms within a number of places e.g. purse/wallet, coat pockets, bags (as well as the carrier), so that users always have a condom on them whatever they take or wear out.

Component four: Using Condoms – hot tips for putting on condoms

See multimedia appendix 10.

This is a video providing step-by-step instructions on how to put a condom on (using a demonstrator). It features a number of young people all giving their tips and tricks on how to do this (correctly, with ease) so that it is enjoyable/pleasurable for self and partner and part of the flow of sex. It is suggested to users' that their partner will appreciate their ability to apply condoms with ease and in pleasurable way.

Component 5: Discussing Condoms – talking to your partner about condoms

See multimedia appendix 11.

This is a series of videos in which young people talk about ways in which they have brought up condom use with partners in the past and introduced them into sex. They talk about how this went, what worked, what didn't etc. The aim is to help users plan the best time to bring up condom use with their partner, and what to do or say if they resist. It is suggested that having beliefs that stick to and also caring about oneself are attractive qualities.

Component 6: Real Life – real people, real experiences

See multimedia appendix 12.

This is a series of three videos featuring three real couples aged between 18-24 years (two straight and one gay). In the videos, the couples talk candidly about sex, and in particular using condoms during sex. They talk about and demonstrate how they put condoms on; who does it, techniques, how make their use part of sex. The shots of the couples talking are interspersed with scenes of them kissing, touching, removing clothes and having sex. The films are akin to the content of a mainstream 18 rated film. There are no explicit shots of

genitals or penetration. Condoms feature in these shots and the couples are shown communicating about them (verbally/non-verbally).
