# Supplementary material for: An Intervention to Increase Condom Use Among Users of Chlamydia Self-Sampling Websites (Wrapped): Intervention Mapping and Think-Aloud Study
Source: JMIR Form Res. 2019 May 1;3(2):e11242. doi: 10.2196/11242 (PMC6658247; doi:10.2196/11242)
Supplement: Multimedia Appendix 7 [file formative_v3i2e11242_app7.pdf]

wrapped

PROFILE SIGN OUT

SAMPLE PACK | ORDER CONDOMS | CONDOM CARRIER | USING CONDOMS | DISCUSSING CONDOMS | REAL LIFE

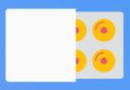

SAMPLE PACK

Here at Wrapped, we're pro condoms, pro pleasure.

But there's nothing pleasurable about a condom that's too tight, slips off, splits or irritates. Maybe the smell, texture, colour or taste also put you off. There's a huge variety out there, but most of us don't get the chance to try them all out. We've put together a selection of condoms and lube so that you can get familiar with what's out there and find the one that works for you. These packs are for everyone – whether you'd wear the condom yourself or put one on someone else – and advice on how to test them out is inside the packs. Go ahead and order your free pack now!

EXAMPLE PACK

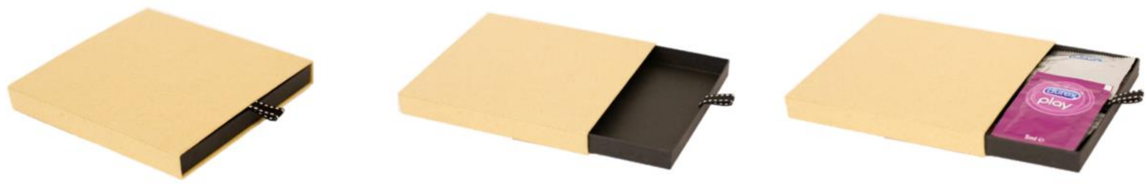

① Design the outside

You have a choice of four options for the box – classic black, natural or a mixture of the two. Which one would you like?

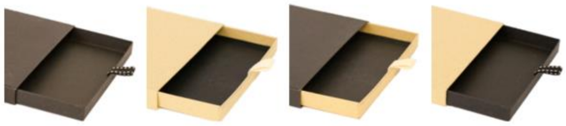

② Design the inside

Choose an image to go inside the box. Which one most reflects your style? Take your pick!

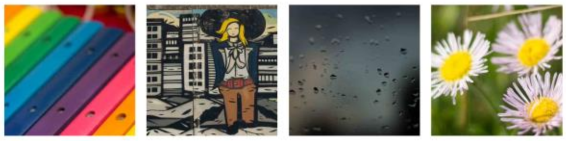

③ Custom condom

If you would like to receive a tailored myONE condom in your pack, please specify a penis size

If you do not enter measurements here we will not be able to include a myONE condom in your sample pack. View measuring guide

Enter Width (mm)

Enter Length (mm)

PROCEED
