# Supplementary material for: An Intervention to Increase Condom Use Among Users of Chlamydia Self-Sampling Websites (Wrapped): Intervention Mapping and Think-Aloud Study
Source: JMIR Form Res. 2019 May 1;3(2):e11242. doi: 10.2196/11242 (PMC6658247; doi:10.2196/11242)

Finding the right condom for you

Not all condoms feel, look, smell and taste the same. Your preference will likely depend on all of these things. The fit is also important; condoms that fit better feel better, and they're also less likely to slip or break. If you have had problems with condoms in the past, don't be put off. This was probably because they didn't fit properly or you needed more practice – read on!

Inside the box are 12 different types of condoms – see the underside of the insert for more info. Over a few days or weeks, take the time to have a look at each in turn. Take them out of the packet and get used to their feel and smell. Have a play around with them and do whatever feels good to you. There are no rights or wrongs here. If you have a penis, try putting the condom on. Alternatively, put one over your fingers or a dildo and then have a play around; try touching yourself and inserting it.

Experiment with some of the lubricant. Move on to masturbating if you like. Focus on pleasurable sensations and how it feels. You'll probably want to lose yourself in the moment, but afterwards try and work out what it was about each condom that you did and didn't like. Try out the different ones and discover your favourite(s).

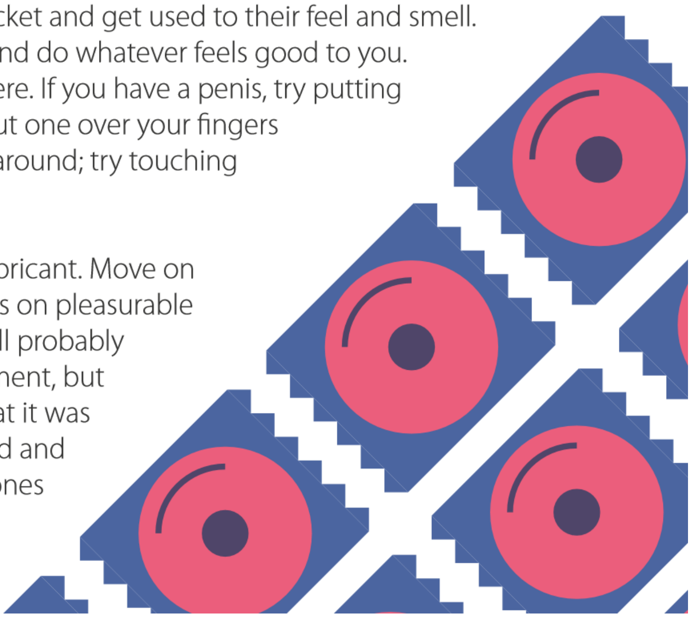

Supplement: Multimedia Appendix 8 [file formative_v3i2e11242_app8.pdf]
