# Supplementary material for: An Intervention to Increase Condom Use Among Users of Chlamydia Self-Sampling Websites (Wrapped): Intervention Mapping and Think-Aloud Study
Source: JMIR Form Res. 2019 May 1;3(2):e11242. doi: 10.2196/11242 (PMC6658247; doi:10.2196/11242)
Supplement: Multimedia Appendix 14 [file formative_v3i2e11242_app14.pdf]

Multimedia appendix 14: Specification of the BCTs and targeted determinants and change objectives for each intervention component

**Intervention component**	**Tailoring**	**Change objective**	**Determinant**	**BCT**
Sample Pack – discover your favourite condom	Not applicable – all users receive this component	Believe that condoms use won't reduce own sexual pleasure	Attitude	Associative learning – condom becomes a conditioned stimulus associated with arousal (conditioned response) as users are encouraged to masturbate with condom and reflect on pleasurable sensations
				Increase positive emotions - Users asked to focus on pleasurable sensations when using condoms
				Behavioural experiments – belief (that sex with condoms is conducive with pleasure) is created through the interpretation of experience when trying out the different condoms
				Re-attribution – explain that condoms which are uncomfortable/reduce

				pleasure are often ill-fitting and therefore users need to spend time finding the right one for them
		Believe that condom use won't reduce spontaneity (impulsiveness and naturalness)	Attitude	Behavioural experiments – knowledge (that can apply condoms with ease thus contributing to naturalness of experience) is created through the interpretation of experience
		Believe that condoms are effective and reliable (won't break, slip etc.) in preventing STIs	Attitude	Re-attribution – explain that condoms which break and slip are usually those which are ill-fitting and that any problems in the past are likely due to that
		Identify preferred type	Behavioural capability	Behavioural experiments – test out different condoms to see which is most comfortable/pleasurable
		Identify place to store supply of condoms	Resources	Adding objects to the environment – new storage for condoms (box)
			Self-efficacy	Instruction on how to perform the behaviour – a

		Believe that can correctly apply and remove		step by step guide to putting on condoms
				Behavioural practice/rehearsal – practice putting on and removing a condom
		Be able to correctly apply and remove	Behavioural capability	Instruction on how to perform the behaviour – step by step guide to putting on condoms
				Behavioural practice/rehearsal – practice putting on and removing a condom
				Framing/re-framing – suggest that being able to put on a condom with ease is a sign of sexual confidence/experience
				Information about others' approval – tell users that partners will find their ability to apply the condom with ease and in a pleasurable way attractive
	I can't always get the type of	Identify where and how can access	Behavioural capability	Instruction on how to perform the behaviour –

Order Condoms – order free condoms	condoms I want; I find condoms expensive to buy; I find buying condoms embarrassing			the Order Condoms service is intuitive/easy to use. Link to other sources of condoms in local area provided
		Plan where and how to access	Behavioural capability	Action planning – encourage users to plan where will get supply of condoms from. NB users sent email reminders to order condoms
		Identify or develop a service to enable easy access to condoms	Resources	Adding objects to the environment – provide access to the Order Condoms service (and signpost to other ways that can access condoms in the local area)
		Identify or develop service that provides a choice of condoms	Resources	Adding objects to the environment – provide access to the Order Condoms service that enables users to try out a range of condoms and then order their preferred type
		Believe that able to buy/request	Self-efficacy	Adding objects to the environment – the Order

				Condoms service removes barriers to accessing condoms (i.e. removes the need for face-to-face request and therefore reduces embarrassment). Self-efficacy for performance of the behaviour should therefore be increased
		Monitor condom supply and obtain more before running out	Behavioural capability	Prompts/cues – message at bottom of box cues the user to order more condoms when running low
		Recognise that others in their peer group use condoms	Perceived norms	Social comparison – demonstrate that other young people use condoms:<br><br>reviews of condoms left by users on the Order Condoms section
Condom Carrier – stash your stuff in this handy free case	I don't always have a condom on me when its needed	Identify a place to carry condom (that will ensure always available)	Resources	Adding objects to the environment – provide users with a product that allows them to discretely carry condom at all times

		Replace used condom asap after use	Behavioural capability	Prompts/cues – note in carrier – ‘replace me!’
		Believe that condom use won’t reduce spontaneity	Attitude	Adding objects to the environment – condoms will always be to hand whenever need them
				Information about social and environmental consequences – users told that having a condom to hand increases spontaneity of sex as can then have sex and time/place of choosing rather than having to first find/access condom
Using Condoms – hot tips for putting on condoms	Condoms make sex less enjoyable or pleasurable for me; Condoms make sex less enjoyable pleasurable for the person I’m with	Believe that condom use won’t reduce own sexual pleasure	Attitude	Information about social and environmental consequences – told about ways in which can apply condom that is conducive to own pleasure
		Believe that condom use won’t reduce partner’s sexual pleasure	Attitude	Information about social and environmental consequences – told about ways in which can apply

	I find using condoms interrupts the flow of sex; I'm not always able to put a condom on with confidence and ease			condom that is conducive to partner's pleasure
		Believe that sex can be enjoyable with condoms	Attitude	Information about social and environmental consequences – told about ways in which can apply condom that is conducive to enjoyment as a couple
		Believe that condom use won't reduce spontaneity	Attitude	Information about social and environmental consequences – told about ways in which can introduce condoms so that experience is natural
		Believe that can correctly apply and remove	Self-efficacy	Instruction on how to perform behaviour – users told how to apply condom
				Demonstration of behaviour – users shown how to put on and remove a condom during the demo video
Discussing Condoms – talking to your	I find using condoms interrupts the flow of sex; I	Believe that condom use won't reduce spontaneity	Attitude	Information about social and environmental consequences – told about ways in which can

partner about condoms	find it awkward or difficult letting someone know that I want to use condoms			introduce condoms so that experience is natural
		Know the best time(s) to make partner aware of intention	Behavioural capability	Instruction on how to perform behaviour – other young people talk about best ways to let their partner know they want to use condoms
		Identify cues that indicate latest point at which could communicate intention	Behavioural capability	Instruction on how to perform behaviour – other young people talk about cues which they use to prompt communication
		Use cues to identify latest point at which could communicate intention	Behavioural capability	Instruction on how to perform behaviour – other young people talk about what they think is the latest point at which could communicate
		Believe that can communicate intention	Self-efficacy	Instruction on how to perform behaviour – other young people talk about ways in which they have let their partner know they want to use condoms
		Believe that can persist with communication in	Self-efficacy	Instruction on how to perform behaviour – others talk about ways in

		the face of resistance (and be prepared to not have sex)		which they have coped with resistance
		Know what will say and/or do to communicate intention	Behavioural capability	Instruction on how to perform behaviour – other young people talk about ways in which they have let their partner know they want to use condoms
		Know what will say/or do if partner disagrees/ refuses	Behavioural capability	Instruction on how to perform behaviour – others talk about what they have said/done when faced with resistance
		Recognise that others in their peer group use condoms	Perceived norms	Social comparison – demonstrate that other young people use condoms
		Believe that can correctly apply and remove	Self-efficacy	Information about others' approval – tell users that partners will respect their ability to apply the condom with ease and in a pleasurable way
		Believe that condom use won't	Attitude	Framing/re-framing – suggest that caring about oneself and doing what is

		reduce partner's sexual pleasure		best for oneself are attractive qualities
Real Life - real people, real experiences	Condoms make sex less enjoyable or pleasurable for me; Condoms make sex less enjoyable pleasurable for the person I'm with; I find using condoms interrupts the flow of sex; I find using condoms a turn-off	Believe that condom use won't reduce own sexual pleasure	Attitude	Associative learning – condom becomes a conditioned stimulus associated with arousal (conditioned response) as users are shown couples having pleasurable sex with condoms<br><br>Vicarious consequences – arousal of individuals is shown in videos
		Believe that condom use won't reduce partner's sexual pleasure	Attitude	Vicarious consequences – arousal of individuals is shown in videos
		Believe that sex can be enjoyable with condoms	Attitude	Vicarious consequences – enjoyment of individuals is shown in videos
		Believe that condom use won't reduce spontaneity	Attitude	Vicarious consequences – using condoms in a natural and spontaneous way is shown in videos

		Believe that can correctly apply and remove	Self-efficacy	Demonstration of behaviour – users shown how to put on and remove a condom during the videos
